# Pilot study of duloxetine plus aerobic exercise for pain and depressive symptoms in knee osteoarthritis

**DOI:** 10.1093/rap/rkag116

**Published:** 2026-10-06

**Authors:** Alan M Rathbun, Alice S Ryan, Yu Dong, Brock A Beamer, Mark Luborsky, Michelle D Shardell, Justine Golden, Claudia M Campbell, Grace H Lo, Jason E Peer, Marc C Hochberg

**Affiliations:** Department of Epidemiology and Public Health, University of Maryland School of Medicine, Baltimore, MD, USA; Department of Medicine, University of Maryland School of Medicine, Baltimore, MD, USA; Department of Medicine, University of Maryland School of Medicine, Baltimore, MD, USA; Geriatric Research Education and Clinical Center, VA Maryland Health Care System, Baltimore, MD, USA; Department of Psychiatry, University of Maryland School of Medicine, Baltimore, MD, USA; Department of Medicine, University of Maryland School of Medicine, Baltimore, MD, USA; Geriatric Research Education and Clinical Center, VA Maryland Health Care System, Baltimore, MD, USA; Institute of Gerontology, Wayne State University, Detroit, MI, USA; Department of Epidemiology and Public Health, University of Maryland School of Medicine, Baltimore, MD, USA; Institute for Genome Sciences, University of Maryland School of Medicine, Baltimore, MD, USA; Department of Epidemiology and Public Health, University of Maryland School of Medicine, Baltimore, MD, USA; Department of Psychiatry and Behavioral Sciences, Johns Hopkins University School of Medicine, Baltimore, MD, USA; Department of Medicine, Baylor College of Medicine, Houston, TX, USA; Department of Psychiatry, University of Maryland School of Medicine, Baltimore, MD, USA; Mental Health Clinical Center, VA Maryland Health Care System, Baltimore, MD, USA; Department of Medicine, University of Maryland School of Medicine, Baltimore, MD, USA

**Keywords:** osteoarthritis, depression, comorbidity/multimorbidity, interventional studies, ageing population

## Abstract

**Objective:**

Pain and depressive symptoms are common in knee osteoarthritis (KOA), and their co-occurrence creates bidirectional effects that complicate treatment, as intervening on one does not necessarily improve the other. This single-group pilot study evaluated the feasibility of a 12-week supervised hybrid aerobic exercise program plus duloxetine targeting pain, depressive symptoms and physical function.

**Methods:**

Between May 2023 and March 2025, participants with symptomatic KOA and mild–severe depressive symptoms were recruited from the University of Maryland Medical System, the VA Maryland Health Care System and the Baltimore metropolitan area. The intervention combined a hybrid walking program (two home-based and one supervised session per week) with duloxetine, initiated at 30 mg and titrated to 60 mg. Clinical outcomes were collected at baseline and 12 weeks and included structured assessments and physical performance tests.

**Results:**

The recruitment target was 20 participants; 34 enrolled, 21 initiated treatment and 17 completed the protocol. Knee Injury and Osteoarthritis Outcome Score pain scores increased by 14.2 points (95% CI 6.5, 21.9), indicating less pain, while Hamilton Depression Rating Scale scores decreased by 5.5 points (95% CI −9.0, −2.0), implying a reduction in depressive symptoms. Physical function improved as measured by changes in the 6-min walk test [130 ft (95% CI 56.7, 204.1)], 20-m gait speed [0.08 m/s (95% CI 0.004, 0.154)] and chair stands [1.8 repetitions (95% CI 0.7, 2.8)].

**Conclusion:**

This study demonstrates feasibility and preliminary efficacy signals for an exercise–pharmacologic protocol and underscores the need for larger randomized clinical trials.

**Clinical Trial Registration:**

https://clinicaltrials.gov/, NCT04111627

Key messages

- First phenotype-targeted KOA intervention combining exercise and pharmacotherapy to treat pain and depressive symptoms.
- Results demonstrate feasibility and large within-participant effects supporting a synergistic care model.
- Findings support larger trials to assess efficacy, clarify effects and identify modifiers and mechanisms.

## Introduction

Knee osteoarthritis (KOA) is a chronic condition that has been classified as a ‘serious disease’ by the US Food and Drug Administration (FDA) because of substantial unmet medical care needs for this patient population [1]. Structural disease and clinical symptoms in KOA develop on a progression trajectory, and despite the enormous public health burden, there are no FDA-approved medications that have been demonstrated to arrest, slow or reverse joint damage [2]. The lack of disease-modifying drugs may be attributed to the heterogeneity of the condition’s pathophysiology that has made it difficult to accurately target pathways for pharmacologic intervention [1, 2]. Accordingly, the most common and safest treatments for symptoms are usual care and/or self-management strategies that are only moderately effective [3]. Given that many patients with KOA have other chronic conditions, greater effort is required to develop improved treatment strategies to address the co-occurrence of arthritis and medical comorbidity.

Symptomatic pain from KOA affects 30% of Americans ≥60 years of age and is a leading cause of functional decline [4]. Moreover, pain management is central to the lived experience of KOA, but patients consistently report that psychological factors shape their perspectives and contribute to their symptoms [5, 6]. As KOA is clinically heterogeneous, empirically derived phenotypes may be targeted with tailored interventions [7]. The most consistently identified phenotype in KOA encompasses pain and depressive symptoms, and up to 25% of individuals meet screening criteria for case-level mood disorder [8, 9]. Previous studies have also shown that persistent depression exacerbates pain severity and accelerates declines in physical function [8, 10, 11]. Despite the need for interventions targeting co-occurring pain and depressive symptoms, current KOA treatment guidelines lack such recommendations, highlighting the need for phenotype-targeted approaches [12].

Aerobic exercise is a first-line treatment modality for KOA management that offers both pain-relieving properties and mood-enhancing benefits [13]. Furthermore, feasibility research implies that patients with KOA and depressive symptoms prefer interventions with home-based components for better accessibility [14]. However, depressive symptoms alter pain perception and hinder aerobic exercise, and as adherence is critical to efficacy, these patients need support to initiate and sustain participation [14]. While aerobic exercise benefits both pain and mood disorders, it is adjunctive to antidepressants when treating depression [15]. As both an analgesic and antidepressant, duloxetine is well-suited for combining with aerobic exercise to enhance adherence and improve overall intervention efficacy [14]. Thus this single-group pilot study tested the feasibility of a 12-week supervised hybrid aerobic exercise plus duloxetine intervention in patients with symptomatic KOA and depressive symptoms. We hypothesized that this multimodal intervention would be feasible and yield preliminary clinical signals regarding pain, depressive symptoms and physical performance.

## Patients and methods

### Study design and sample

This single-arm, open-label pilot study evaluated the feasibility and preliminary efficacy signals of multimodal intervention using a 12-week hybrid supervised aerobic exercise program plus duloxetine and assessed logistical parameters needed to plan a larger comparative efficacy trial. Research indicates that ≥12 participants are needed per group for pilot studies and that precision in variance estimates improves as sample sizes approach 20 [16]. Thus a target sample of 20 completers provides reasonably stable estimates of variability and effect sizes in a single-arm design. Progression to a randomized controlled trial (RCT) would be supported if at least 75% of participants initiating the intervention completed the 12-week protocol, and additional secondary benchmarks included ≥75% adherence to each intervention component, including centre-based exercise, home-based exercise and duloxetine [17]. Ethical study procedures were reviewed and approved by the University of Maryland, Baltimore Institutional Review Board (HP-00089160). All study participants provided written informed consent and all study procedures were followed in accordance with the ethical standards of the responsible committee on human experimentation (institutional and national) and with the Helsinki Declaration of 1975, as revised in 2000.

Participants with symptomatic KOA and depressive symptoms were recruited via electronic medical records and digital advertisements. Patients from the University of Maryland Medical System and the VA Maryland Health Care System were identified through EPIC and the VISN 5 Corporate Data Warehouse using International Classification of Diseases, Ninth/Tenth Revision codes 715.96/M17, respectively, and study advertisements were disseminated on platforms such as Facebook and Google. Eligibility criteria were age ≥40 years, English speaking, symptomatic KOA consistent with American College of Rheumatology criteria [18], no planned knee surgery within 3 months, mild to severe depressive symptoms on the 9-Item Patient Health Questionnaire (PHQ-9 score ≥5) [19] and the ability to participate in supervised aerobic exercise. Exclusion criteria were any exercising two or more times per week; current use of duloxetine, antipsychotics, benzodiazepines or opioid analgesics; cognitive impairment on the Mini-Mental State Examination (MMSE score <20) [20]; past or current bipolar disorder or psychosis per the Diagnostic and Statistical Manual of Mental Disorders, Fifth Edition [21]; substance use disorder or suicidal ideation in the previous year; significant cardiovascular, pulmonary or peripheral vascular disease limiting safe exercise; active chemotherapy or radiation; and pregnancy or lactation.

### Screening and enrolment

Interested individuals completed an initial telephone prescreening to confirm KOA symptoms, depressive symptom eligibility, medication exclusions and safety for aerobic exercise and duloxetine. At the baseline visit, written informed consent was obtained and depressive symptoms were reassessed. Cognitive screening and structured psychiatric assessment were also performed to rule out disqualifying comorbid diagnoses. A medical history and physical examination with safety laboratory testing were used to confirm eligibility and clearance for aerobic exercise and duloxetine initiation. Individuals who did not meet eligibility criteria were restricted from further participation and were provided with appropriate clinical resources.

### Multimodal intervention

The aerobic exercise component consisted of a 12-week hybrid walking program using one supervised session per week and two home-based sessions per week. Centre-based sessions were supervised by exercise physiologists. Exercise intensity targets were individualized using peak heart rate from a graded exercise test performed prior to starting the program and prescribed using heart rate reserve (HRR; Karvonen formula) [22]. Sessions began with a goal of 15 min at 40–50% HRR, with intermittent treadmill walking and rest intervals permitted as needed. Treadmill parameters (speed, incline and duration) were progressed as tolerated toward 60–75% HRR and 50 min/session [23]. Heart rate and blood pressure were assessed before and after supervised sessions. Blood glucose was also assessed for participants with diabetes, and perceived exertion was recorded after each supervised session. Participants wore a Fitbit device (www.fitbit.com) with heart rate monitoring and completed exercise logs. Fitbit data were synced to a study account accessible to study staff to monitor adherence and fidelity and to verify self-reported exercise logs. Home-based walking followed the same progression targets as supervised sessions and could be completed in multiple bouts on a treadmill or in the community.

Duloxetine was initiated at 30 mg once daily, and after a 2-week titration period, increased to 60 mg once daily as tolerated for the remainder of the 12-week intervention. Medication was managed by the study psychiatrist with monitoring for tolerability and adverse events throughout the program. Participants taking other antidepressants at enrolment completed a clinician-supervised taper prior to duloxetine initiation when clinically appropriate. Dose reductions or temporary holds were permitted for side effects and all medication changes were recorded. Every 2 weeks, participants completed structured telephone calls to monitor medication adherence and assess potential adverse events, which were also assessed during centre-based exercise sessions.

### Measures

Self-reported demographic and socio-economic factors and clinical characteristics were assessed at enrolment. Study outcomes were assessed at baseline (post-enrolment, pretreatment) and at the 12-week follow-up. The two co-primary outcomes were pain severity, measured using the Knee injury and Osteoarthritis Outcome Score (KOOS) pain subscale, and depressive symptoms, assessed using the Hamilton Depression Rating Scale (HAM-D) [24, 25]. Consistent with recommended outcomes for non-operative KOA trials, secondary outcomes included physical function measured using the 6-min walk test (6MWT), 20-m walk time (20mWT), 30-s chair stand test (30CST) and timed up and go (TUG) [26]. Exploratory outcomes were knee-related health status (other KOOS subscales), anxiety [General Anxiety Disorder-7 (GAD-7)], fatigue (PROMIS Fatigue SF-8a), sleep impact (ASCQ-Me Sleep Impact–Short Form), self-efficacy (PROMIS Self-Efficacy SF-8a) and pain catastrophizing [Pain Catastrophizing Scale (PCS)].

### Statistical analyses

Baseline characteristics were summarized among enrolled participants (*N* = 34). Continuous measures were described using means and standard deviations, and categorical variables using frequencies and percentages. Feasibility metrics included recruitment, enrolment and retention; descriptive intervention uptake and adherence; and safety and tolerability based on adverse events. Given the single-group, open-label design, participants who initiated the intervention (*n* = 21) served as their own controls over the 12-week study period. Box plots were used to visualize pre- and post-intervention values for the co-primary outcomes (pain severity and depressive symptoms). Within-person changes from baseline to 12 weeks were estimated using paired *t*-tests among participants with non-missing baseline and 12-week outcome data (*n* = 17) to assess whether the mean change differed from zero. Given the single-arm design, analyses emphasized descriptive estimation and paired comparisons rather than formal modelling. Results are reported as marginal mean values at baseline and the 12-week follow-up, with corresponding within-person changes presented with 95% CIs and paired-data effect sizes (ESs).

## Results

### Recruitment

Recruitment and retention are shown in Fig. 1. A total of 515 individuals expressed interest and 105 completed telephone prescreening. Among prescreened participants, 34 individuals were subsequently enrolled. There were 13 enrolled participants who did not complete baseline data collection (7 became ineligible and 6 withdrew prior to or during baseline assessments), leaving 21 participants who initiated the intervention. Retention after treatment initiation was high: only four participants did not complete the 12-week assessment (one was lost to follow-up, one withdrew, one was restricted from participation due to safety concerns and one was discontinued due to protocol non-adherence), resulting in 17 participants who completed the study per protocol.

**Figure 1:**
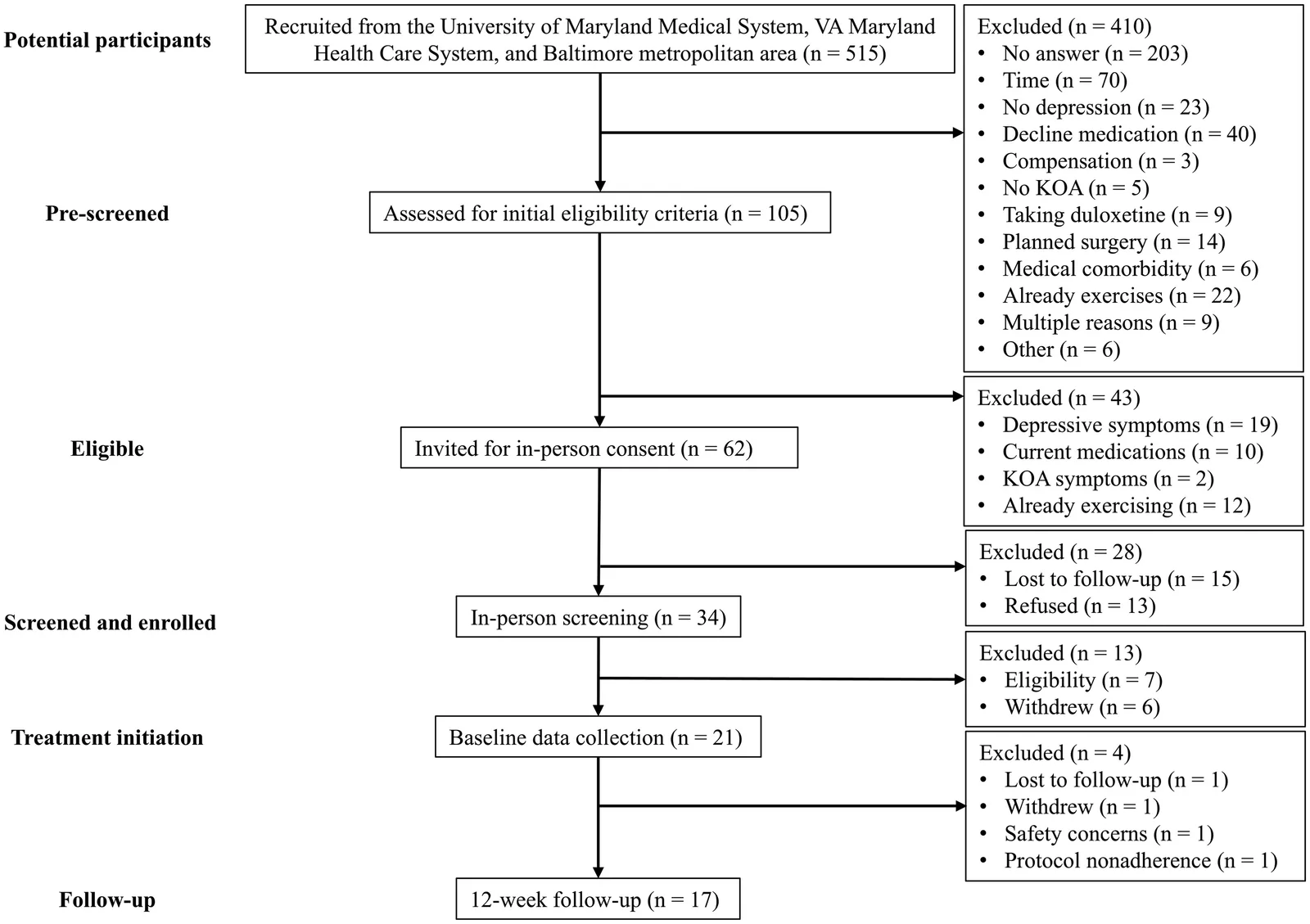
Study sample flow diagram. Multiple reasons indicates participants for whom more than one of the listed exclusion reasons applied. Other represents participants whose exclusion reason could not be classified into one of the predefined categories

### Sample characteristics

The study sample was composed primarily of older females (mean age 61.4 years) who identified as non-Hispanic White or African American (Table 1). Approximately one-fifth of participants were veterans (21.9%) and marital status was distributed across never married and married (31.2% each), divorced (21.9%) and widowed (15.6%). Most participants had some form of employment and at least some college or professional education beyond high school. The majority also had health insurance and reported using prescription medications, over-the-counter medications or supplements. Enrolled participants generally reported severe or very severe knee pain and depressive symptoms, with a mean PHQ-9 score of 13.8, meeting the screening threshold for major depression (PHQ-9 score ≥10).

**Table 1:** Baseline study sample characteristics.

Variable	Mean	s.d.	*n*	%
Age, years	61.38	10.17		
Female			22	64.7
Race				
Black			16	47.1
White			16	47.1
Other			2	5.9
Hispanic			2	5.9
Veteran			7	21.9
Marital status				
Never			10	31.2
Married			10	31.2
Divorced			7	21.9
Widowed			5	15.6
Education level				
High school graduate			4	12.9
Vocational			1	3.2
Some college			7	22.6
Associates			4	12.9
Some college or professional school			2	6.5
College graduate			6	19.4
Master’s			6	19.4
Doctorate			1	3.2
Employment status				
Unemployed			3	9.7
Full time			15	48.4
Part time			2	6.5
Retired			5	16.1
Semiretired			2	6.5
Disability			4	12.9
Health insurance			28	82.4
Knee pain severity				
Moderate			11	32.4
Severe			18	52.9
Very severe			5	14.7
PHQ-9 score	13.79	3.53		
Prescription medications			32	94.1
OTC medications			30	88.2
Supplements			25	73.5

Percentages were calculated using available nonmissing observations and may reflect different denominators across variables.

### Adherence, safety and clinical outcomes

Medication adherence to the duloxetine component was extremely high (>95%). Adherence to the aerobic exercise component was lower, with participants completing 79.4% of centre-based sessions and 68.5% of prescribed home-based exercise among all who initiated the intervention (*n* = 21). Adherence was higher among participants who completed the intervention (*n* = 17) and reached 90.7% and 79.4%, respectively. There were 12 adverse events during the study period and none of them were serious. Six adverse events were potentially related to the intervention and primarily involved mild medication-related symptoms. Participants who completed the 12-week multimodal intervention showed statistically significant within-person reductions in both pain severity and depressive symptoms (Fig. 2). The average KOOS pain and HAM-D scores were 50.49 and 15.18 at baseline and 64.71 and 9.65 at the 12-week follow-up, respectively. Marginal within-participant changes in these outcomes were estimated to be +14.22 points (95% CI 6.49, 21.94; *P* = 0.001; ES = 0.946) for KOOS pain and −5.53 points (95% CI −9.04, −2.02; *P* = 0.004; ES = 0.811) for HAM-D.

**Figure 2:**
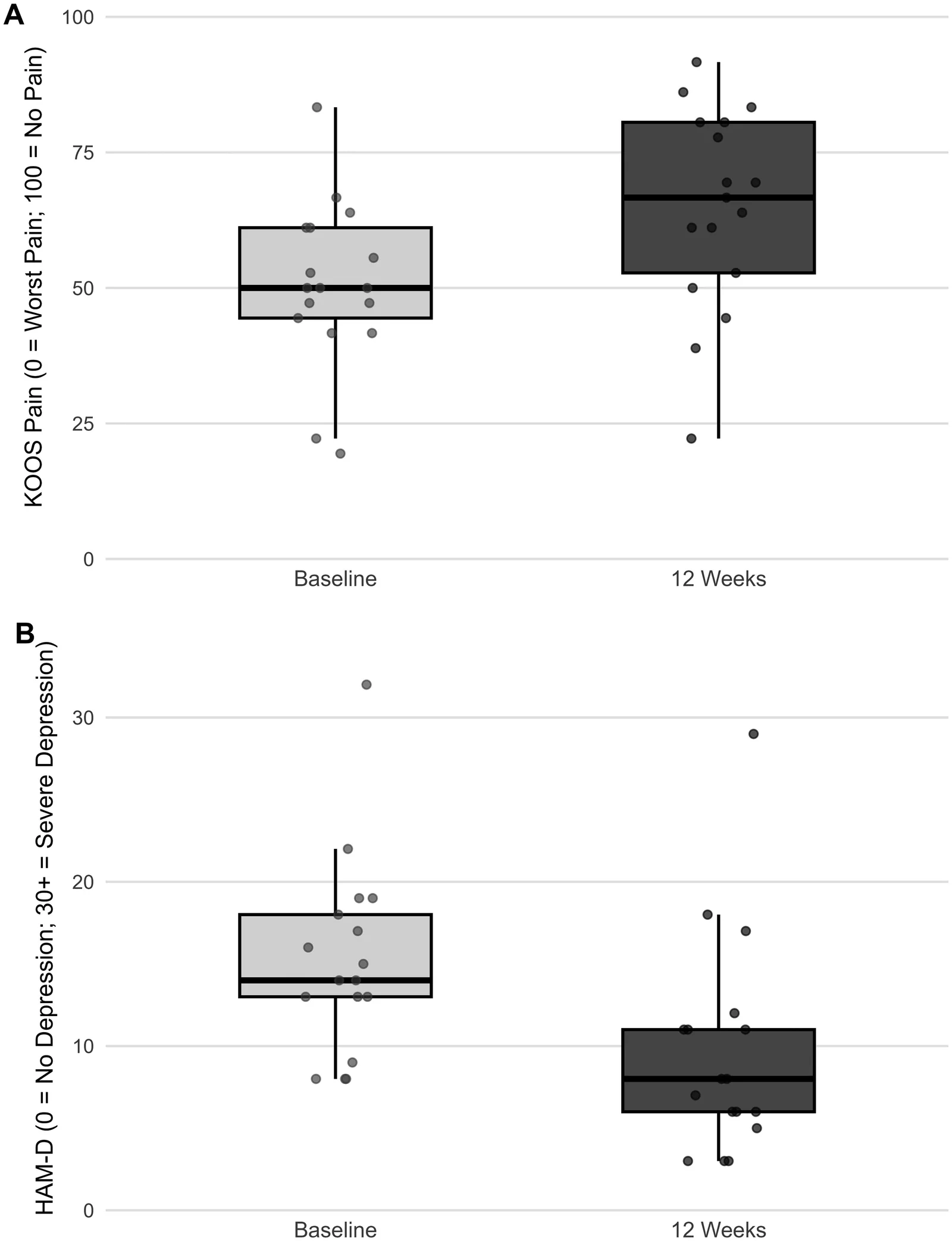
Changes in pain severity and depressive symptoms from baseline to 12 weeks. **(A)** KOOS pain subscale; **(B)** HAM-D

### Secondary and exploratory outcomes

Participants also showed statistically significant within-person changes in secondary physical performance measures and other exploratory patient-reported outcomes (Table 2). The 6MWT increased by 130.4 feet on average and 20mWT decreased by 1.26 s, corresponding to an increase in gait speed of 0.079 m/s. Performance on the 30CST also improved by an average of 1.77 repetitions. These objective improvements were accompanied by significant self-reported gains on the KOOS activities of daily living (ADL) subscale (+15.1 points). Statistically significant reductions in psychological parameters were observed, including anxiety (Δ = –4.53) and pain catastrophizing (Δ = –9.00). These improvements in physical performance and psychological health were accompanied by reduced fatigue (Δ = –9.97) and increased self-efficacy (Δ = 6.22) and quality of life (Δ = 15.07).

**Table 2:** Within-participant changes in physical performance and patient-reported outcomes from baseline to 12 weeks follow-up (*n* = 17).

Outcome	Baseline	Follow-up	**Δ**	95% CI	ES	*P*-value
20mWT, s	19.50	18.23	−1.26	−2.22, −0.31	−0.68	0.013
6MWT, feet	1268.53	1398.94	130.41	56.74, 204.08	0.91	0.002
30CST, repetitions	9.94	11.71	1.77	0.73, 2.80	0.88	0.002
Gait speed, m/s	1.08	1.16	0.08	0.01, 0.15	0.55	0.039
TUG, s	11.48	11.74	0.26	−0.98, 1.49	0.11	0.666
Sleep impact (PROMIS T-score)	54.91	56.76	1.85	−2.97, 6.66	0.20	0.428
Fatigue (PROMIS T-score)	59.49	49.53	−9.97	−14.62, −5.31	−1.10	<0.001
GAD-7 (0–21)	8.29	3.77	−4.53	−6.65, −2.41	−1.10	<0.001
KOOS ADL (0–100)	54.67	69.72	15.05	7.49, 22.61	1.02	0.001
KOOS QOL (0–100)	22.79	37.87	15.07	3.21, 26.94	0.65	0.016
KOOS sport/rec (0–100)	15.29	28.53	13.24	−2.87, 29.34	0.42	0.101
KOOS symptoms (0–100)	52.31	64.92	12.61	5.67, 19.54	0.93	0.001
Pain catastrophizing (0–52)	21.35	12.35	−9.00	−14.51, −3.49	−0.84	0.003
Self-efficacy (PROMIS T-score)	46.95	53.17	6.22	2.17, 10.27	0.79	0.005

Δ: 12-week change; QOL: quality of life; sport/rec: sports and recreation.

## Discussion

This study tested the feasibility of a multimodal intervention combining scalable treatment components with complementary mechanisms of action to reduce pain severity and depressive symptoms and improve physical function to mitigate functional decline among individuals with symptomatic KOA. Recruitment demonstrated strong interest among individuals with symptomatic KOA and depressive symptoms and supports the feasibility of delivering this intervention. Preliminary clinical signals also included reductions in pain severity and depressive symptoms that suggest this phenotype-targeted intervention could be efficacious in a common KOA subgroup. These improvements were accompanied by increases in walking speed, endurance, lower extremity strength and psychological health and quality of life.

Prior interventional studies in patients with KOA and major depression largely reflect routine unimodal clinical care models, emphasizing improved access to care, stepped antidepressant or psychotherapy approaches or education and self-management, while leaving physical activity largely unaddressed [27–30]. Moreover, treatment efficacy among patients with KOA and depression is limited when interventions target either condition in isolation [31–33]. In contrast, our intervention was grounded in observational research showing that depressive symptoms contribute to bidirectional dynamics in KOA, with physical function, depression and pain severity exacerbating one another over time, forming a symptomatic feedback loop (physical function ⇄ depression ⇄ pain) [11, 34, 35]. Furthermore, separate mechanistic and pharmacoepidemiologic studies highlighted the need for treatments that address both conditions in parallel and demonstrated greater effectiveness of oral analgesics with increasing persistence of pain and depressive symptoms [10, 36]. These data motivated our feasibility study testing a 24-week centre-based aerobic exercise program combined with duloxetine; however, we identified additional challenges related to accessibility, including time commitment and mood disorder case definition [14]. Collectively, this evidence formed the foundation for the current 12-week hybrid aerobic exercise plus duloxetine intervention administered to patients with symptomatic KOA across a spectrum of depressive symptom severity.

Feasibility was supported by recruitment and retention, while clinical outcomes provided preliminary efficacy signals for this exercise–pharmacologic protocol. For example, recruitment in this study exceeded that of a prior multisite, single-group pilot intervention targeting physical and emotional pain in KOA and major depression, in which 14 participants were enrolled and 13 completed the protocol [28]. Similarly, the number of individuals who expressed interest in participation was ≈70% greater (≈500 *vs* ≈300) than that reported in an RCT assessing internet-based cognitive behavioural therapy for patients with KOA and major depression [30]. Within-person changes in pain severity and depressive symptoms were large (ES ≥0.8) and comparable to prior interventional studies [27–30]. Notably, prior studies enrolled patients with KOA and case-level mood disorder, who typically have more severe pain and depression and a larger opportunity for treatment response, whereas our sample included individuals across the full spectrum of depressive symptoms. Thus our findings suggest comparable preliminary clinical signals in a more heterogeneous population and further support evaluation of this phenotype-targeted intervention to prevent progression of KOA symptoms and the onset of major depression. More importantly, our intervention used an exercise program focused on functional improvement, augmented with targeted pharmacologic treatment, and was associated with statistically significant gains across multiple objective physical performance outcomes. In contrast, prior intervention models prioritize pharmacologic and psychological care optimization and rarely incorporate objective measures of physical function when assessing treatment response [27–30]. Nonetheless, opportunities remain to further enhance the accessibility, scalability and efficacy of this intervention through continued refinement and evaluation in future studies.

There are multiple strengths of this study. Foremost, our exercise–pharmacologic intervention leveraged extensive observational and feasibility research and integrates phenotype-targeted yet scalable complementary treatments with cross-condition effects designed to optimize adherence and potential efficacy. Second, the study design used an enrichment approach to recruit KOA participants with clinically relevant depressive symptoms rather than restricting eligibility based on diagnostic criteria for major depression. The intervention also incorporated a hybrid delivery model, combining supervised gym-based sessions with home-based exercise, supported by medical and psychiatric monitoring to ensure safety, progression and adherence. We recruited participants from the University of Maryland Medical System, the VA Maryland Health Care System and the broader Baltimore metropolitan area to enhance the generalizability of our findings. Lastly, study outcomes included objective measures of physical performance, including gait speed, endurance and lower extremity strength, which have rarely been assessed in prior interventions targeting KOA and depression.

Nevertheless, findings must be interpreted within the context of study limitations. The single-group design precludes attributing the observed preliminary clinical signals to the intervention and does not provide estimates of treatment efficacy. Clinical outcome analyses were restricted to participants who completed the 12-week intervention, which may overestimate preliminary clinical signals due to potential selection bias. Alternatively, the observed changes in clinical outcomes may reflect regression to the mean, contextual or Hawthorne effects or natural variation over time. The geographically and subgroup-specific sample may limit the generalizability of recruitment, retention, adherence and other feasibility findings to more heterogeneous populations within the targeted KOA depressive symptom phenotype. Although the prespecified progression criterion requiring 75% completion of the 12-week protocol among participants who initiated the intervention was satisfied, additional intervention development and evaluation in a RCT are needed to establish efficacy. Despite these limitations, the findings provide preliminary evidence supporting the feasibility and potential efficacy of a scalable and multimodal intervention for patients with KOA and depressive symptoms.

In conclusion, a hybrid aerobic exercise program combining supervised centre-based and home-based sessions, augmented with duloxetine pharmacotherapy, was feasible among individuals with symptomatic KOA across a range of depressive symptom severity. This multimodal intervention also yielded preliminary efficacy signals in pain severity, depressive symptoms, physical performance and other psychological and quality-of-life outcomes. These findings support progression to further evaluation in larger multi-arm RCTs to assess the individual and combined effects of the intervention components. Lastly, identifying clinically relevant mechanisms and moderators of treatment response will clarify who benefits most and why.

## Data Availability

The data underlying this article are available from the corresponding author upon reasonable request and subject to institutional and ethical approvals.
